# Vaccination and Vaccine Effectiveness: A Commentary of Special Issue Editors

**DOI:** 10.3390/vaccines8030545

**Published:** 2020-09-18

**Authors:** Claudio Costantino, Alessandra Casuccio, Vincenzo Restivo

**Affiliations:** Department of Health Promotion, Maternal and Infant Care, Internal Medicine and Medical Specialties “G. D’Alessandro”, Section of Hygiene, University of Palermo, Via Del Vespro 133, 90127 Palermo, Italy; alessandra.casuccio@unipa.it (A.C.); vincenzo.restivo@unipa.it (V.R.)

**Keywords:** vaccination, vaccine effectiveness, vaccine hesitancy, healthcare workers, influenza vaccination, hepatitis B vaccination, human papillomavirus vaccination, measles, mumps, rubella and varicella vaccination, herpes zoster vaccination, internationally adopted children

## Abstract

The Special Issue “Vaccination and Vaccine Effectiveness”, published in the journal *Vaccines*, has the main aim to increase international literature data on vaccine effectiveness and safety and on vaccination strategies in order to reduce vaccine hesitancy and improve vaccination coverage rates. The main topics included in the call for papers were vaccines administered to infants, adolescents, adults, elderly people, at-risk populations (due to comorbidities and personal risk factors) and healthcare workers and strategies adopted to promote vaccination adherence among these categories. This Special Issue started from the assumption that, despite vaccination being universally recognized as one of the best strategies to increase duration and quality of life during the last centuries, vaccination coverage rates are often under the levels recommended to reduce circulation and to extinguish vaccine-preventable diseases. Vaccine hesitancy involves at least 15% of the general population, and healthcare workers also sometimes demonstrate doubts on vaccination effectiveness and safety. At the end of the six-month submission period, 16 articles (15 research article and one review) were accepted after the peer-review processes and published online.

## 1. Background

Vaccination is universally recognized as one of the best strategies to increase duration and quality of life during the last centuries [1]. Nevertheless, vaccination coverage rates are under the levels recommended to limit spread and to reduce the burden on health systems of vaccine-preventable diseases [2,3,4].

In several countries, vaccination acceptance is low due to misconceptions or doubt about the effectiveness and safety of vaccines [5,6]. The vaccine hesitancy involves at least 15% of the general population, and healthcare workers also sometimes demonstrate doubts on vaccination [7,8].

A Special Issue entitled “Vaccination and Vaccine Effectiveness” was launched by the journal *Vaccines* in June 2019 with the main aim of collecting international literature data on vaccine effectiveness and safety and on strategies that could contribute to reducing vaccine hesitancy and improving vaccination coverage rates [9].

Submissions of original articles, systematic reviews or meta-analyses, short communications and other types of article on vaccines administered to infants, adolescents, adults, elderly people, at-risk populations (due to comorbidities and personal risk factors) and healthcare workers and strategies adopted to promote vaccination adherence among these categories were welcomed and encouraged for this Special Issue.

## 2. Manuscripts Included in the Special Issue

At the end of December 2019, 16 manuscripts were submitted and, after the peer-review process, accepted for publication in the Special Issue (SI) “Vaccination and Vaccine Effectiveness” of *Vaccines*. In particular, 15 original articles (research articles) and one review article were published online in the final Special Issue.

Several topics were addressed in the SI “Vaccination and Vaccine Effectiveness”, and all manuscripts published and available online in open-access form are reported in chronological publishing order in Table 1 with the following main characteristics: authorship (first author), topic, location where research were conducted, timeframe, methodology and main findings of the study.

Pacenti et al. analyzed the seroprevalence of measles and vaccination coverage rates in the Veneto Region, Italy, from 2009 to 2017 among the general population and observed that measles outbreaks were common especially in areas with suboptimal vaccine uptake [10].

At the University Hospital (UH) of Palermo, Italy, a quasi-experimental field trial was conducted from 2007 to 2019 in order to evaluate vaccination increase during consecutive influenza seasons among health care workers (HCWs) of the UH of Palermo after communicative and informative tailored strategies were adopted, and the impact of this increase on the reduction of working days lost due to acute sickness during influenza season [11].

Piazza et al. conducted a retrospective cohort study on hospitalizations due to herpes zoster in Liguria Region, Italy, highlighting the considerable burden of this disease among elderly and frailpeople [12]. Nicoli and colleagues realized a multicenter observational study in Italy among women previously vaccinated against HPV and observed a good humoral (IgG) and cellular (B cell-mediated) response over time [13].

An observational study conducted by Cassimos et al. analyzed vaccination policies in 42 European countries, evidencing significant differences nowadays in vaccination programs for adults among the different countries considered in the study [14].

Another study conducted in Sicily, Italy, evaluated the impact of HPV universal vaccine introduction into the vaccination schedule ten years ago, through the analysis of hospital discharge records. A significant decrease in hospital admissions for cervical cancer and other HPV-related diseases emerged after HPV vaccine introduction in Sicily, despite not satisfying vaccination coverage rates [15].

Bechini and colleagues evidenced that more than half of internationally adopted children who visited Meyer Children’s University Hospital in Florence, Italy, between 2009 and 2018, if aged 15–18 years and originating from Africa, were not protected against MMRV [16]. Mascia et al. demonstrated in a multicenter survey that Italian adolescents share information and knowledge on vaccination especially through social networks [17]. Furthermore, from a comparison between two of the most populous (Emilia Romagna and Sicily) Italian regions, it emerged that the legislative coercive measures applied in Italy at the end of 2017 had a favorable impact on MMRV vaccination coverage rates among children [18].

Among IAC with an unknown protective status against MMRV, as reported by Boccalini et al., a vaccination strategy based on serotesting results was the most advantageous approach, economically and clinically [19]. Moreover, another multicenter study conducted ina Northern (Liguria) and a Southern (Apulia) Italian region found poor knowledge but good attitudes among undergraduates about HPV vaccination [20]. A review conducted on immunization protocols of IAC in Western countries demonstrated substantially different approaches that should be corrected [21]. Glatman-Freedman et al. analyzed the variation in the vaccine effectiveness (VE) of influenza vaccination in Israel during the 2018/2019 season according to different clades of influenza A (H3N2) virus [22], and Barbara et al. observed a substantial increase of vaccination adherence among HCWs of the Catholic UH of Rome, Italy, due to evidence-based promoting strategies [23]. Panatto and colleagues, through a case-control test negative design, analyzed the VE of influenza vaccination during the 2018/2019 season in two Italian regions [24]. Finally, a study on HBV seroprevalence in pediatric and adolescent populations of Tuscany, Italy, found that immunity due to HBV vaccination reduced virus circulation [25].

## 3. Conclusions

One of the main challenges for public health, particularly for vaccinology, in the next decades is the contrast in vaccine hesitancy among the general population. In response to this phenomena, strong multidisciplinary alliances between health care professionals, providing evidence-based data on vaccine effectiveness and safety and on reduction of the burden of vaccine-preventable disease due to vaccinations offered, addressing vaccine hesitancy through innovative communication strategies and increasing opportunities to administer vaccines among the general population (at school, at work, at the hospital ward) are some of the possible strategies that should be considered by international and national health authorities and agencies [26,27,28].

## Figures and Tables

**Table 1:** Description of the manuscripts accepted for the Special Issue “Vaccination and Vaccine Effectiveness” in chronological publishing order.

Authorship	Research Topic	Location	Timeframe	Study Methodology	Main Findings
Pacenti et al. [10]	Measles vaccination uptake and seroprevalence	Veneto Region (Italy)	2009–2012	Cross-sectional study	Measles outbreaks are common in areas with suboptimal vaccine coverage rates
			2014–2017		
Costantino et al. [11]	Influenza vaccination adherence among Health care Workers (HCWs)	University Hospital of Palermo, Sicily, Italy	2007–2019	Quasi-experimental field trial	Increasing influenza vaccination coverage rates among HCWs could reduce working days lost due to acute sickness during influenza season
Piazza et al. [12]	Burden of hospitalization due to herpes zoster	Liguria Region, Italy	2015–2017	Retrospective cohort study	Herpes zoster causes a considerable burden of disease among elderly and frail people
Nicoli et al. [13]	Antibody and B cell response to HPV vaccines	Veneto and Emilia Romagna Regions, Italy	2018–2019	Observational study	HPV vaccines guarantee good humoral (IgG) and cellular (B cell-mediated) response
Cassimos et al. [14]	Vaccination policies for adults in Europe	42 European countries	2019	Observational study	Significant differences in vaccination programs for adults are observed among European countries
Restivo et al. [15]	HPV-related hospitalizations ten years after universal vaccine introduction	Sicilian Region, Italy	2007–2017	Retrospective observational study	A decrease in hospital admissions for cervical cancer and other HPV-related diseases emerged after HPV vaccine introduction
Bechini et al. [16]	Immunization status against measles, mumps, rubella and varicella (MMRV) in internationally adopted children (IAC)	Meyer Children’s University Hospital, Florence, Italy	2009–2018	Retrospective observational study	More than half of IAC (especially if aged 15–18 years and originating from Africa) are not protected against MMRV
Mascia et al. [17]	Adolescent vaccination behavior	Central Italy	2016–2018	Cross-sectional study	Adolescents share information and knowledge on vaccination through social networks
Gori et al. [18]	Impact of mandatory vaccination on MMRV vaccine adherence	Emilia Romagna and Sicilian Regions, Italy	2009–2018	Retrospective observational study	Legislative coercive measures applied in Italy have a favorable impact on MMRV vaccination coverage rates
Boccalini et al. [19]	Clinical and economic impact of MMRV vaccination on IAC	Meyer Children’s University Hospital, Florence, Italy	2009–2018	Retrospective observational study	MMRV vaccination based on serotesting results is the most advantageous strategy for IAC
Trucchi et al. [20]	HPV vaccine knowledge and attitudes among young adults	Liguria and Apulia Regions, Italy	2017–2018	Cross-sectional study	Poor knowledge and good attitudes were found among undergraduates about HPV vaccination
Alimenti et al. [21]	Immunization protocols of IAC in Western countries	France, Italy, Spain, Ireland, UK, USA, Canada	2004–2019	Review	Substantially different approaches to immunization protocols of IAC children have emerged in Western countries
Glatman-Freedman et al. [22]	Drifted influenza A (H3N2) clade in influenza season 2018/2019	Israel Influenza Surveillance Network	2018–2019	Observational study	Vaccine effectiveness of influenza vaccination varies according to different clades of influenza A (H3N2) virus
Barbara et al. [23]	Influenza vaccination coverage rates among HCWs	Catholic University Hospital of Rome, Italy	2015–2019	Quasi-experimental field trial	Vaccination adherence among HCWs increased after evidence-based promoting strategies
Panatto et al. [24]	Influenza vaccination effectiveness (VE) and adherence among HCWs	Lombardy and Liguria Regions, Italy	2018–2019	Case-control test negative and retrospective cohort studies	Influenza vaccination VE during 2018/2019 was below 50%, and vaccination adherence could limit influenza.
Zanella et al. [25]	Hepatitis B virus (HBV) seroprevalence in pediatric and adolescent populations	Tuscany Region, Italy	2017–2018	Observational study	HBV seroprevalence highlights that immunity due to universal vaccination reduced circulation of the virus in pediatric and adolescent populations
